# Cardiorespiratory Response to Exercise in Parkinson's Disease: Associations with Autonomic Dysfunction and Physical Activity

**DOI:** 10.1002/mdc3.70172

**Published:** 2025-06-09

**Authors:** Kars I. Veldkamp, Sabine Schootemeijer, Hilde Joosten, Bastiaan R. Bloem, Luc J.W. Evers, Nienke M. de Vries

**Affiliations:** ^1^ Radboud University Medical Center, Donders Institute for Brain, Cognition and Behaviour, Department of Neurology Center of Expertise for Parkinson and Movement Disorders Nijmegen the Netherlands; ^2^ Department of Sports Medicine Canisius Wilhelmina Hospital Nijmegen the Netherlands; ^3^ Department of Human Movement Sciences University Medical Center Groningen Groningen the Netherlands

**Keywords:** Parkinson's disease, cardiorespiratory fitness, physical functioning, exercise, autonomic dysfunction

## Abstract

**Background:**

Both autonomic dysfunction and low levels of physical activity could contribute to reduced cardiorespiratory fitness in people with Parkinson's disease (PD). However, the interrelationship between these concepts is not well understood.

**Objectives:**

Our aim was to gain more insight into autonomic dysfunction and physical activity in relation to cardiorespiratory fitness in PD.

**Methods:**

We included 59 individuals with PD (37% women, mean age 65.1 years; mean disease duration 4.5 years; Hoehn and Yahr stage 1–3). All participants completed a cardiopulmonary exercise test (CPET). We examined the association between parameters assessed during a CPET (peak oxygen consumption [VO_2peak_], maximum heart rate [HR_max_], heart rate recovery at 1 and 3 mins [HR_rec1_, HR_rec3_]), autonomic dysfunction (Scales for Outcomes in Parkinson's disease‐Autonomic dysfunction, SCOPA‐AUT), and physical activity in daily life (step counts, measured using a smartphone for a period of 4 weeks). Using multivariable regression, we adjusted for age, sex, and beta blocker use.

**Results:**

Higher SCOPA‐AUT was associated with lower HR_max_ (*β* = −0.72, 95% CI = [−1.40, −0.04], *P* = 0.040). Higher step counts were associated with higher HR_rec3_ (*β* = 3.21, 95% CI = [0.56, 5.86], *P* = 0.019), and with higher VO_2peak_ (*β* = 1.16, 95% CI = [0.10, 2.22], *P* = 0.032). No statistically significant associations were found between both SCOPA‐AUT and step counts with HR_rec1_.

**Conclusions:**

Both autonomic dysfunction and physical activity are associated with a reduced cardiorespiratory response to exercise in PD, but affected different CPET parameters. Future studies should determine the responsiveness of these CPET parameters to change, in order to implement such parameters as endpoints in clinical trials.

Higher cardiorespiratory fitness is associated with lower mortality and morbidity in the general population.^1^ Cardiorespiratory fitness could be lower in people with Parkinson's disease (PD).^2^, ^3^, ^4^ Moreover, cardiorespiratory fitness might also be associated with disease severity.^5^, ^6^ Multiple factors could play a role in mediating cardiorespiratory fitness in PD and in this study we further explore two of these. First, autonomic dysfunction, an early non‐motor sign of PD, may contribute to a reduced cardiorespiratory fitness in people with PD.^2^ As a result of parasympathetic and sympathetic denervation, the heart rate response to exercise could be dysregulated.^3^ This dysregulation contributes to a reduced maximum heart rate during exercise, possibly resulting in a reduced maximal oxygen consumption.^7^ Second, people with PD are physically less active than healthy older adults,^8^ which is also associated with a reduced cardiorespiratory fitness in people with PD.^9^

Exercise interventions are often evaluated with a cardiopulmonary exercise test. Previous exercise interventions in PD reported peak oxygen consumption and maximum heart rate.^5^, ^10^, ^11^ Heart rate recovery, the change in heart rate after stopping with exercise, can be influenced by autonomic function^12^ and physical activity.^13^ Additional parameters such as heart rate recovery, which have not been extensively reported in PD, could provide a more comprehensive insight into the cardiorespiratory response to exercise in people with PD.

In this study, we investigate the association between a comprehensive set of parameters assessed with a cardiopulmonary exercise test (CPET) and measures of autonomic dysfunction on the one hand, and physical activity on the other hand. We hypothesized that more autonomic dysfunction would be associated with a reduced maximum heart rate, and that higher levels of physical activity would be associated with a higher peak oxygen consumption. For heart rate recovery, we hypothesized that more physical activity would be associated with better heart rate recovery. Deeper understanding of CPET parameters could support their use as endpoints in clinical trials.

## Methods

### Participants and Setting

We analyzed the baseline data from a subset of participants (n = 71) who enrolled in the STEPWISE study (NCT04848077) between July 2021 and March 2024. STEPWISE is a randomized controlled trial investigating whether using a motivational smartphone app for 1 year can increase physical activity in people with PD.^14^ To be included in STEPWISE, participants had to have PD confirmed by a neurologist, Hoehn and Yahr stage 1–3, able to understand Dutch, able to walk independently, perform ≤120 min of sports‐ or outdoor activities per day and take an average of <7000 steps per day during a 4‐week screening period. Participants were excluded if they experienced weekly falls in the 3 months prior to screening, had medical conditions that hamper mobility other than PD, were living in a nursing home, had cognitive impairments that hamper use of the STEPWISE app or were not in the possession of a suitable smartphone (iPhone 5S or newer with iOS10 or higher or Android 4.1 or newer). The PD diagnosis was verified by letters from the neurologist to the general practitioner. Until March 2024, 320 participants were enrolled in the STEPWISE study. We studied the 71 participants in the STEPWISE study that performed a CPET. Enrollment for the CPET was pragmatic, based on availability of the CPET and participant. Participants were screened for cardiovascular risk by the sports physicians before participation in the CPET.

The protocol for the STEPWISE study was approved by the medical ethical committee of Arnhem‐Nijmegen (NL75501.091.20). Informed consent was signed during the baseline visit for the study. STEPWISE is conducted at Radboud University Medical Center (Radboudumc) Nijmegen, CPETs were administered at Canisius Wilhelmina Ziekenhuis (CWZ).

### Study Procedures

Participants were screened on baseline physical activity (step counts) using the STEPWISE app on participants’ smartphone for 4 weeks. If participants were deemed eligible (≤7000 steps), they completed a set of baseline assessments at Radboudumc after which they started with the intervention. Participants’ height, weight, age, years of education, disease duration and smoking status were collected in‐clinic at the Radboudumc. Participants self‐administered questionnaires on autonomic dysfunction (SCales for Outcomes in PArkinson's disease‐Autonomic Dysfunction; SCOPA‐AUT)^15^ and non‐motor aspects of experiences in daily living (Movement Disorders Society‐Unified Parkinson's Disease Rating Scale; MDS‐UPDRS I)^16^ online through our data management system (CastorEDC)^17^ at home. PD motor symptoms (MDS‐UPDRS III)^16^ were assessed in clinic. Participants underwent all assessments in their regular medicated state and assessments were conducted by trained researchers. After these assessments, participants completed a CPET at the CWZ.

### Cardiopulmonary Exercise Test

A CPET is the gold standard to assess cardiorespiratory fitness and was conducted on a cycle ergometer (Lode, Excalibur sport) by sports physicians at the CWZ. Gas exchange was collected breath‐by‐breath with a Vyntus™ CPX metabolic cart. Data were analyzed per eight breaths on a rolling average with SentrySuite™ software. Participants were instructed to fast for 1.5 h before the CPET and to refrain from caffeine or carbonated drinks on the day of the CPET. Before the test, maximal exercise capacity was estimated by the sports physician based on questions on exercise habits and age. A ramp protocol was used, in which the maximal effort was achieved between 8 and 12 min. Before the CPET started, participants performed a 3‐min warm‐up on the cycle ergometer, set at a load of 0 Watt, and at a cadence of 70–80 revolutions per minute. Sports physicians provided auditory cues to help the participant to reach this cadence if needed and provided verbal support during the test. The CPET was terminated when the participant was not able to keep up the cadence or at the request of the participant. After the test, participants remained seated on the cycle ergometer for 3 min to assess heart rate recovery. Participants performed active recovery during the first 2 min after exertion to reduce the risk of fainting. The resistance during these first 2 min of recovery was dependent on the ramp protocol (eg, for a ramp protocol of 100 Watt the resistance was 10 Watt, for a ramp protocol of 150 Watt, the resistance was 25 Watt). During this active recovery, no instructions were given regarding the cadence. Between 2 and 3 min after test termination, the cadence was gradually reduced to zero revolutions at a set resistance of 0 Watt.

Respiratory exchange ratio (RER) was determined at maximal exertion. Lactate level was determined with the Lactate Pro2 blood lactate meter in the finger at rest and at maximal exertion, as well as the rate of perceived exertion (RPE; BORG scale 6–20).^18^ Heart rate (beats/min) was continuously monitored with the Cardiosoft ECG system. The physician collected the heart rate at rest, at the first and second ventilatory threshold, at maximal exertion and 1 and 3 min after maximal exertion. Heart rate recovery at 1 and 3 min were chosen to cover the parasympathetic reactivation (at 1 min) and the combination of parasympathetic reactivation and sympathetic deactivation (at 3 min).^19^ Oxygen consumption (mL/kg/min) and power (Watt) were collected at baseline, at the first and second ventilatory threshold and at maximal exertion. Use of beta blockers was registered before the test was initiated. Sports physicians entered the results directly into CastorEDC.^17^

Participants were excluded if the sports physician observed cardiac arrythmia during the CPET, because heart rate parameters obtained during cardiac arrythmia are not reflective of autonomic dysfunction. A CPET was considered valid if a plateau in VO_2_ during 30 s was reached, despite a further increase in work rate. However, a CPET was also determined to be a “maximal” effort (and therefore valid) if the RER ≥ 1.00 in combination with an increase in blood lactate level from pre‐ to post‐test by 4.0 mmol/L or if it exceeded 8.0 mmol/L at test termination. Two supportive measures were used: the age‐predicted maximal heart rate ≥ 90% and a post‐test RPE ≥ 16.

### Variables of Interest

For the cardiorespiratory response to exercise, we studied heart rate recovery at 1 min (HR_rec1_) and 3 min (HR_rec3_), maximum heart rate (HR_max_) and peak oxygen consumption (VO_2peak_). We report the highest measured heart rate (HR_max_) and oxygen consumption (VO_2peak_) during the test.

For autonomic dysfunction, we used the total score of the SCOPA‐AUT questionnaire (higher values meaning more symptoms). For physical activity, we studied the step count collected via the STEPWISE app, which participants installed on their own smartphone. Step counts were collected locally using the HealthKit (iPhone) and Google Fit (Android) platforms. We used step counts collected over a 4‐week period, which served as the baseline step count assessment for the STEPWISE trial.^14^ During this baseline period, participants could only their cumulative step counts.

### Statistics

We explored the relationship between each dependent variable (HR_rec1_, HR_rec3_, HR_max_ and VO2_peak_) and autonomic dysfunction (SCOPA‐AUT) and physical activity (step count) with multivariable linear regression (Fig. S1). Using multivariable regression, we adjusted for age, sex, and beta blocker usage. In the analysis with step counts, betas were calculated per 1000 steps. We calculated the partial variance that was explained by each independent variable. Nominal *P*‐values were reported and considered statistically significant when lower than 0.05. Analyses were performed in R (RStudio, version 4.3.2).

### Sensitivity Analyses

In the Supplement, we show the following sensitivity analyses: (i) quantifying autonomic dysfunction by the self‐reported first factor of the MDS‐UPDRS part I (non‐motor aspects of experiences in daily living) instead of the SCOPA‐AUT^16^; (ii) quantifying autonomic dysfunction by the sum of items 14–16 and 14–21 on the SCOPA‐AUT; (iii) excluding three participants on beta blockers instead of using beta blockers as covariate in the model; (iv) adding levodopa equivalent daily dosage (LEDD) as covariate in the model; (v) a multivariable regression analysis with SCOPA‐AUT as independent variable and RER, blood lactate and RPE as dependent variables.

## Results

### Demographics

From the 70 participants in STEPWISE that performed a CPET, we excluded two participants because of arrythmia (atrial flutter and atrial fibrillation). From the 68 participants with a CPET that remained, we included 59 participants with a valid test according to the sports physician (Table 1).

**TABLE 1:** Descriptive characteristics for total sample and split for valid and invalid cardiopulmonary exercise test according to the sports physician

	Total (n = 68)	Valid (n = 59)	Invalid (n = 9)
Age [years]	65.6 (7.7)	65.1 (7.9)	69.3 (5.3)
Women	26 (38.2%)	22 (37.3%)	4 (44.4%)
Body mass index [kg/m^2^]	26.9 (3.9)	26.7 (3.9)	28.2 (4.1)
Education [years]	14.0 [12.0–18.0]	14.0 [12.0–18.0]	14.9 [12.0–18.0]
Disease duration [years]	3.5 [1.0–6.0]	4.0 [1.5–6.0]	3.0 [1.0–6.0]
Symptom onset [years]^a^	6.0 [3.8–12.0]	6.5 [4.0–12.0]	5.0 [3.5–14.0]
Hoehn and Yahr stage^a^			
1	30 (44.1%)	27 (45.8%)	3 (33.3%)
2	27 (39.7%)	22 (37.3%)	5 (55.6%)
3	8 (11.8%)	7 (11.9%)	1 (11.1%)
LEDD [mg]	575 [378–810]	600 [389–818]	405 [300–575]
SCOPA‐aut score	16.0 (7.3)	16.3 (7.0)	14.3 (9.1)
VO_2peak_ [mL/kg/min]	24.6 (6.2)	25.3 (6.1)	20.0 (5.2)
HR_max_ [bpm]	134.8 (21.6)	137.9 (20.5)	114.8 (18.3)
Respiratory exchange ratio	1.12 (0.10)	1.15 (0.08)	0.99 (0.07)
MDS‐UPDRS‐III score	31.2 (11.8)	30.7 (11.4)	34.7 (13.9)
Six minute walking distance [m]	489.5 (78.5)	494.3 (75.5)	457.7 (95.0)
Step counts [steps/day]	4410.4 (1441.0)	4518.2 (1384.5)	3703.6 (1687.1)
Current smoker	1 (1.5%)	1 (1.7%)	0 (0%)
Use of beta blockers	3 (4.3%)	3 (5.1%)	0 (0%)

*Note*: Mean (standard deviation), median [interquartile range] or frequency (%) is reported. Hoehn and Yahr stage was missing for three participants that performed a valid test.

Abbreviations: HR_max_, maximum heart rate; LEDD, levodopa equivalent daily dosage; MDS‐UPDRS, movement disorders society‐unified Parkinson disease rating scale; SCOPA, scales for outcomes in Parkinson's disease‐autonomic dysfunction; VO_2peak_, peak oxygen consumption.

^a^ Data on symptom onset was missing for one participant that performed a valid test. Data on Hoehn and Yahr stage was missing for three participants that performed a valid test.

Participants with a valid test had a mean age of 65 (standard deviation, SD: 7.9) years, 22 (37%) were women, they were diagnosed with PD 4.5 (SD: 3.5) years ago and had a baseline physical activity level of 4518 (SD: 1384.5) steps per day (Table 1). Four participants were referred to a cardiologist after the CPET for a check‐up.

### Autonomic Function

**Figure 1:**
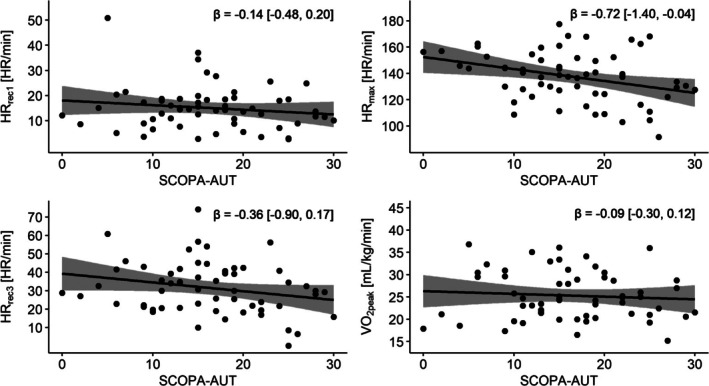
Multivariable regression results of CPET parameters and autonomic dysfunction. (A) Heart rate recovery 1 min (HR_rec1_). (B) Maximum heart rate (HR_max_). (C) Heart rate recovery 3 min post exercise (HR_rec3_). (D) Peak oxygen consumption (VO_2peak_). We report the beta‐coefficients and 95% confidence intervals. Each data point was corrected for the following covariates: age, sex, use of beta blockers, and step count. CPET, cardiopulmonary exercise test; SCOPA‐AUT, scales for outcomes in Parkinson's disease‐autonomic dysfunction.

Higher SCOPA‐AUT was associated with lower HR_max_ (SCOPA‐AUT; *β* = −0.72, 95% CI = [−1.40, −0.04], *P* = 0.040; Fig. 1; Table 2). No statistically significant associations were found between SCOPA‐AUT and VO_2peak_, HR_rec1_, and HR_rec3_.

**TABLE 2:** Univariable and multivariable regression analyses including the CPET parameters (HR_rec1_, HR_rec3_, HR_max_ and VO_2peak_) as dependent variables and SCOPA‐AUT, step counts, age, sex and beta blocker usage as independent variables

Dependent variable	Independent variable	*β*‐coefficient (SE)	95% CI	*P*‐value	Adj *r* ^2^ (*r* ^2^)	Partial *r* ^2^
HR_rec1_	SCOPA‐AUT	−0.21 (0.17)	[−0.54, 0.12]	0.211	0.01 (0.027)	NA
HR_rec1_	Step counts	1.15 (0.84)	[−0.54, 2.84]	0.177	0.015 (0.032)	NA
HR_rec1_	SCOPA‐AUT	−0.14 (0.17)	[−0.48, 0.20]	0.416	0.022 (0.107)	0.013
	Step counts	1.18 (0.85)	[−0.52, 2.88]	0.169		
	Age	−0.06 (0.15)	[−0.37, 0.24]	0.676		
	Sex	−3.97 (2.53)	[−9.05, 1.12]	0.123		
	Beta blocker use	0.77 (5.44)	[−10.15, 11.68]	0.889		
HR_rec3_	SCOPA‐AUT	−0.53 (0.28)	[−1.09, 0.03]	0.065	0.042 (0.059)	NA
HR_rec3_	Step counts	3.26 (1.4)	[0.45, 6.06]	0.024	0.07 (0.087)	NA
HR_rec3_	SCOPA‐AUT	−0.36 (0.27)	[−0.90, 0.17]	0.176	0.187 (0.257)	0.034
	Step counts	3.21 (1.32)	[0.56, 5.86]	0.019		
	Age	−0.52 (0.24)	[−1.00, −0.05]	0.032		
	Sex	−5.37 (3.96)	[−13.31, 2.56]	0.180		
	Beta blocker use	−4.99 (8.50)	[−22.04, 12.07]	0.560		
HR_max_	SCOPA‐AUT	−0.96 (0.36)	[−1.69, −0.23]	0.011	0.094 (0.109)	NA
HR_max_	Step counts	2.92 (1.92)	[−0.93, 6.77]	0.134	0.022 (0.039)	NA
HR_max_	SCOPA‐AUT	−0.72 (0.34)	[−1.40, −0.04]	0.040	0.247 (0.312)	0.077
	Step counts	2.87 (1.70)	[−0.54, 6.28]	0.097		
	Age	−0.65 (0.31)	[−1.26, −0.04]	0.038		
	Sex	−12.11 (5.09)	[−22.33, −1.9]	0.021		
	Beta blocker use	−3.57 (10.94)	[−25.51, 18.37]	0.746		
VO_2peak_	SCOPA‐AUT	−0.09 (0.11)	[−0.32, 0.14]	0.449	−0.007 (0.01)	NA
VO_2peak_	Step counts	1.27 (0.56)	[0.15, 2.39]	0.027	0.067 (0.083)	NA
VO_2peak_	SCOPA‐AUT	−0.09 (0.11)	[−0.30, 0.12]	0.400	0.185 (0.255)	0.013
	Step counts	1.16 (0.53)	[0.10, 2.22]	0.032		
	Age	−0.31 (0.09)	[−0.50, −0.12]	0.002		
	Sex	2.74 (1.58)	[−0.43, 5.91]	0.089		
	Beta blocker use	−0.84 (3.40)	[−7.65, 5.97]	0.806		

*Note*: Betas for step counts should be interpreted per 1000 steps.

Abbreviations: CPET, cardiopulmonary exercise test; HR_max_, maximum heart rate; HR_rec1_, heart rate 1 min posttest termination; HR_rec3_, heart rate 3 min posttest termination; SCOPA‐AUT, scales for outcomes in Parkinson's disease‐autonomic dysfunction; VO_2peak_, peak oxygen consumption.

### Physical Activity

**Figure 2:**
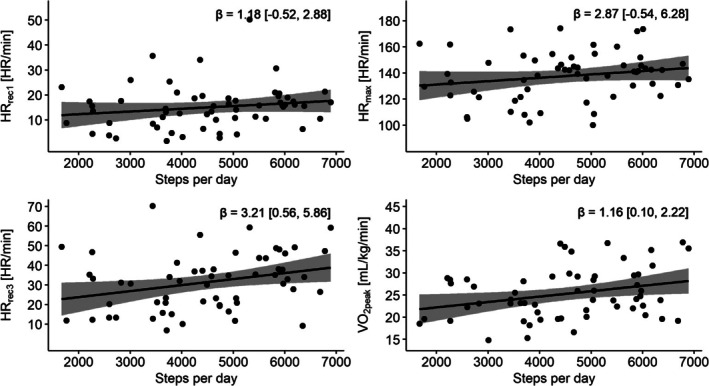
Multivariable regression results of CPET parameters and physical activity. (A) Heart rate recovery 1 min (HR_rec1_). (B) Maximum heart rate (HR_max_). (C) Heart rate recovery 3 min post exercise (HR_rec3_). (D) Peak oxygen consumption (VO_2peak_). We report the beta‐coefficients and 95% confidence intervals. Each data point was corrected for the following covariates: age, sex, use of beta blockers, and SCOPA‐AUT (in the regression for step counts). Betas should be interpreted per 1000 steps. CPET, cardiopulmonary exercise test; SCOPA‐AUT, scales for outcomes in Parkinson's disease‐autonomic dysfunction.

Higher step counts were associated with higher HR_rec3_ (*β* = 3.21, 95% CI = [0.56, 5.86], *P* = 0.019) and with higher VO_2peak_ (*β* = 1.16, 95% CI = [0.10, 2.22], *P* = 0.032; Fig. 2; Table 2). No statistically significant associations were found between step count and HR_rec1_ and HR_max_.

### Sensitivity Analyses

For the sensitivity analyses, we found (i) similar associations for MDS‐UPDRS I.I, although none of the associations remained statistically significant (Data S1, Table S1 and Fig. S2); (ii) no statistically significant associations for the SCOPA items 14–16 and 14–21 (Figs. S3 and S4); (iii) unchanged associations after excluding three participants on beta blockers instead of using beta blockers as a covariate in the model (Figs. S5 and S6); (iv) unchanged associations after adding LEDD as covariate in the model (Figs. S7 and S8), although the association between SCOPA‐AUT and HR_max_ did not remain statistically significant; (v) no statistically significant associations between SCOPA‐AUT and RER, blood lactate and RPE at maximal exertion (Fig. S9).

## Discussion

We demonstrated that both autonomic dysfunction and physical inactivity are associated with reduced cardiorespiratory response to exercise in PD, but that each of these factors may affect different CPET parameters. Greater autonomic dysfunction was associated with a lower maximum heart rate. Higher levels of physical activity were associated with higher heart rate recovery at 3 min and with higher peak oxygen consumption.

In line with our hypothesis, more autonomic dysfunction was associated with lower maximum heart rate. Chronotropic incompetence, a reduced heart rate response during maximal exercise, can be the result of autonomic dysfunction. Others showed that chronotropic incompetence is common in people with PD performing a cardiorespiratory fitness test.^20^ Autonomic dysfunction was not associated with heart rate recovery. Maximum heart rate is mainly driven by sympathetic activation, whilst heart rate recovery is predominantly regulated by parasympathetic reactivation.^19^ Since we only found an association between autonomic dysfunction and maximum heart rate, and not with heart rate recovery, this might indicate that people with more autonomic dysfunction may have more dysfunction of cardiac sympathetic activity. However, we did not find evidence for an association between CPET parameters and the cardiovascular items of the SCOPA‐AUT. This inconsistency with the analysis on the SCOPA‐AUT total score may be due to the fact that orthostatic hypotension (SCOPA items 14–16) and thermoregulatory‐ and pupillomotor problems (SCOPA items 17–21) are experienced later (up to 10 years) after PD diagnosis.^21^, ^22^ Our cohort of people with PD with a mean disease duration of 4.5 years, may already show subtle distortions in heart regulation due to autonomic dysfunction, which are not yet picked up by these SCOPA‐AUT sub scores. Furthermore, autonomic dysfunction was not associated with peak oxygen consumption. This seems surprising, especially since we show an association between autonomic dysfunction and maximum heart rate. Based on Fick's principle, a reduced cardiac output (as result of a reduced maximum heart rate) would result in a reduced oxygen consumption.^7^ Possible compensating mechanisms are increases in blood volume or ventricular compliance,^23^ but these are hypothetical and warrant further study.

We found that higher levels of physical activity were associated with higher heart rate recovery at 3 min and higher peak oxygen consumption. The association between physical activity and peak oxygen consumption is in line with a previous study in 113 people with PD^9^ and in healthy older adults.^24^ Although we cannot draw causal conclusions from our observational data, the association observed here suggests that increasing step counts could increase peak oxygen consumption as well. Increasing step counts may be an accessible, relatively easy, intervention, potentially resulting in improved cardiorespiratory fitness.

The positive association between step count and VO_2peak_, highlights the potential for improving cardiorespiratory fitness in individuals with PD. Notably, the lack of an association between autonomic dysfunction and VO_2peak_ suggests that even those people with PD who experience severe autonomic dysfunction may benefit. This highlights the importance of personalized training programs to optimize exercise benefits. Furthermore, both VO_2peak_ and heart recovery parameters should be prioritized in studies that evaluate the efficacy of exercise interventions. Future research should incorporate blood pressure measurements to gain a better insight into autonomic dysfunction. For example, combining the active standing test and blood pressure measurements during exercise, as described in a previous study,^25^ would allow to differentiate between neurogenic orthostatic hypotension and/or exercise induced hypotension.

### Strengths and Limitations

We extend previous studies by including a range of CPET parameters next to peak oxygen consumption, namely heart rate recovery and maximum heart rate. In addition, we carefully considered criteria for evaluating the validity of fitness tests in the PD population. For our study, sport physicians verified the validity of a fitness test. In this assessment, they took into account oxygen consumption, RER, lactate, heart rate response and perceived exertion of the participant. This approach is different from previous proposals.^11^ The main difference is that we used heart rate as supportive measure and did not exclude participants based on a maximum heart rate criterion. This was a deliberate choice, since people with PD may experience chronotropic incompetence,^20^ reflective of autonomic dysfunction. Although we support the effort to harmonize reporting of cardiopulmonary exercise tests, this proposed heart rate criterion would have discarded 28 out of 59 tests analyzed in this study (Tables S2 and S3). Lastly, in comparison to previous studies, we included a large sample of cardiorespiratory fitness tests.^11^

This study was not without shortcomings. First, our sample consists of people with PD who voluntarily participate in a trial aiming to increase physical activity. Moreover, we included only participants that were not already physically active (≤ 7000 steps per day). This could limit the external validity of our findings due to selection bias, although our findings remain indicative for this subpopulation of relatively inactive persons with PD. Second, we analyzed autonomic dysfunction using a self‐reported questionnaire. Some of the questionnaires used in the PD field have a high within‐subject variability and a low test–retest reliability,^26^ although this is not specifically known for the autonomic questionnaire used here. To overcome this, we advocate the use of objective and/or continuous measures of autonomic dysfunction in future research. For example, researchers should consider quantitative methods to assess autonomic function, such as wearable devices to monitor heart rate variability,^27^ the Valsalva maneuver to assess the presence of autonomic dysfunction^28^ or to study cardiac innervation with an imaging modality like 123I‐mIBG scintigraphy.^29^ This would allow to gain a deeper understanding into autonomic dysfunction. Finally, the resistance was standardized during recovery but sports physicians did not instruct participants to follow a specific cadence after termination of the exercise test. This could have influenced the heart rate recovery parameters. In future work, we advise to standardize the cadence during active recovery.

## Conclusion

A better understanding of which CPET parameters respond to training is key for the evaluation of exercise interventions. Our findings suggest to prioritize peak oxygen consumption and heart rate recovery at 3 min over maximum heart rate and heart rate recovery at 1 min. Future studies need to determine the responsiveness of these cardiorespiratory parameters to exercise interventions, in order to implement such parameters as endpoints in clinical trials.

## Author Roles

(1) Research project: A. Conception, B. Organization, C. Execution; (2) Analysis: A. Design, B. Execution, C. Review and Critique; (3) Manuscript: A. Writing of the first draft, B. Review and Critique.

K.V.: 1A, 1B, 1C, 2A, 2B, 3A

S.S.: 1A, 1B, 1C, 2A, 2B, 3A

H.J.: 1A, 2C, 3B

B.B.: 1A, 2C, 3B

L.E.: 1A, 2A, 2C, 3B

N.V.: 1A, 2A, 2C, 3B

## Disclosures

**Ethical Compliance Statement:** We confirm that we have read the Journal's position on issues involved in ethical publication and affirm that this work is consistent with those guidelines. The study was approved by the medical ethical committee of Arnhem‐Nijmegen (NL75501.091.20). Informed consent was signed in two‐fold by the participants and researcher at the Radboudumc. Participants kept one copy.

**Funding Sources and Conflicts of Interest:** No specific funding was received for this work. The STEPWISE study is funded by ZonMw (The Netherlands Organization for Health Research and Development; grant number 546003007). The authors declare that there are no conflicts of interest relevant to this work.

**Financial Disclosures for the Previous 12 Months:** KV was financially supported by the Dutch Research Council Long‐Term Program (project #KICH3.LTP.20.006, financed by the Dutch Research Council, Verily, and the Dutch Ministry of Economic Affairs and Climate Policy). SS was supported by ZonMw (The Netherlands Organization for Health Research and Development; grant number 546003007). L.E. was financially supported by the Dutch Research Council (grant #ASDI.2020.060) and the Ministry of Education, Culture and Science (through the sector plan medical and health sciences). NV was financially supported by ZonMw (grant numbers 91619142 and 546003007), the Michael J. Fox Foundation and Verily Life Sciences. BB currently serves as Editor in Chief for Journal of Parkinson's Disease, serves on the editorial board of Practical Neurology and Digital Biomarkers, has received fees from serving on the scientific advisory board for the Critical Path Institute, Gyenno Science, MedRhythms, UCB, Kyowa Kirin and Zambon (paid to the Institute), has received fees for speaking at conferences from AbbVie, Bial, Biogen, GE Healthcare, Oruen, Roche, UCB and Zambon (paid to the Institute), and has received research support from Biogen, Cure Parkinson's, Davis Phinney Foundation, Edmond J. Safra Foundation, Fred Foundation, Gatsby Foundation, Hersenstichting Nederland, Horizon 2020, IRLAB Therapeutics, Maag Lever Darm Stichting, Michael J. Fox Foundation, Ministry of Agriculture, Ministry of Economic Affairs & Climate Policy, Ministry of Health, Welfare and Sport, Netherlands Organization for Scientific Research (ZonMw), Not Impossible, Parkinson Vereniging, Parkinson's Foundation, Parkinson's UK, Stichting Alkemade‐Keuls, Stichting Parkinson NL, Stichting Woelse Waard, Health Holland/Topsector Life Sciences and Health, UCB, Verily Life Sciences, Roche and Zambon.

## Supporting information


**Data S1.** Methods and results of MDS‐UPDRS I.I as independent variable.

**Figure S1.** Directed Acyclic Graph of the parameters under study. We hypothesized that Parkinson's disease could lead to autonomic dysfunction, and that autonomic dysfunction reduces the cardiorespiratory response to exercise. We also expect that Parkinson's disease reduces physical activity, and that physical activity results in a reduced cardiorespiratory response to exercise.

**Figure S2.** Multivariable regression results of CPET parameters and MDS‐UPRS‐I.I. (A) Heart rate recovery 1 min (HR_rec1_). (B) Maximum heart rate (HR_max_). (C) Heart rate recovery 3 min post exercise (HR_rec3_). (D) Peak oxygen consumption (VO_2peak_). We report the beta‐coefficients and 95% confidence intervals. Each data point was corrected for the following covariates: age, sex, use of beta blockers, and step count (divided by 1000). CPET, cardiopulmonary exercise test; MDS‐UPDRS, movement disorders society‐unified Parkinson disease rating scale.

**Figure S3.** Multivariable regression results of CPET parameters and autonomic dysfunction on items 14–16 of the SCOPA‐AUT. (A) Heart rate recovery 1 min (HR_rec1_). (B) Maximum heart rate (HR_max_). (C) Heart rate recovery 3 min post exercise (HR_rec3_). (D) Peak oxygen consumption (VO_2peak_). We report the beta‐coefficients and 95% confidence intervals. Each data point was corrected for the following covariates: age, sex, use of beta blockers, and step count. CPET, cardiopulmonary exercise test; SCOPA‐AUT, scales for outcomes in Parkinson's disease‐autonomic dysfunction.

**Figure S4.** Multivariable regression results of CPET parameters and autonomic dysfunction on items 14–21 of the SCOPA‐AUT. (A) Heart rate recovery 1 min (HR_rec1_). (B) Maximum heart rate (HR_max_). (C) Heart rate recovery 3 min post exercise (HR_rec3_). (D) Peak oxygen consumption (VO_2peak_). We report the beta‐coefficients and 95% confidence intervals. Each data point was corrected for the following covariates: age, sex, use of beta blockers, and step count. CPET, cardiopulmonary exercise test; SCOPA‐AUT, scales for outcomes in Parkinson's disease‐autonomic dysfunction.

**Figure S5.** Multivariable regression results of CPET parameters and autonomic dysfunction, excluding three participants on beta blockers. (A) Heart rate recovery 1 min (HR_rec1_). (B) Maximum heart rate (HR_max_). (C) Heart rate recovery 3 min post exercise (HR_rec3_). (D) Peak oxygen consumption (VO_2peak_). We report the beta‐coefficients and 95% confidence intervals. Each data point was corrected for the following covariates: age, sex and step count. CPET, cardiopulmonary exercise test; SCOPA‐AUT, scales for outcomes in Parkinson's disease‐autonomic dysfunction.

**Figure S6.** Multivariable regression results of CPET parameters and physical activity, excluding three participants on beta blockers. (A) Heart rate recovery 1 min (HR_rec1_). (B) Maximum heart rate (HR_max_). (C) Heart rate recovery 3 min post exercise (HR_rec3_). (D) Peak oxygen consumption (VO_2peak_). We report the beta‐coefficients and 95% confidence intervals. Each data point was corrected for the following covariates: age, sex and scales for outcomes in Parkinson's disease‐autonomic dysfunction (SCOPA‐AUT). CPET, cardiopulmonary exercise test.

**Figure S7.** Multivariable regression results of CPET parameters and autonomic dysfunction, correcting for levodopa equivalent daily dose. (A) Heart rate recovery 1 min (HR_rec1_). (B) Maximum heart rate (HR_max_). (C) Heart rate recovery 3 min post exercise (HR_rec3_). (D) Peak oxygen consumption (VO_2peak_). We report the beta‐coefficients and 95% confidence intervals. Each data point was corrected for the following covariates: age, sex, step count and levodopa equivalent daily dose. CPET, cardiopulmonary exercise test; SCOPA‐AUT, scales for outcomes in Parkinson's disease‐Autonomic dysfunction.

**Figure S8.** Multivariable regression results of cardiorespiratory parameters and physical activity, correcting for levodopa equivalent daily dose. (A) Heart rate recovery 1 min (HR_rec1_). (B) Maximum heart rate (HR_max_). (C) Heart rate recovery 3 min post exercise (HR_rec3_). (D) Peak oxygen consumption (VO_2peak_). We report the beta‐coefficients and 95% confidence intervals. Each data point was corrected for the following covariates: age, sex, SCOPA‐AUT and levodopa equivalent daily dose. CPET, cardiopulmonary exercise test; SCOPA‐AUT, scales for outcomes in Parkinson's disease‐autonomic dysfunction.

**Figure S9.** Multivariable regression results including respiratory exchange ratio, blood lactate and rate of perceived exertion at test termination as dependent variables and SCOPA‐AUT as independent variable. We report the beta‐coefficients and 95% confidence intervals. Each data point was corrected for the following covariates: age, sex, use of beta blockers, and step count. RER, respiratory exchange ratio; RPE, rate of perceived exertion; SCOPA‐AUT, scales for outcomes in Parkinson's disease‐autonomic dysfunction.

**Table S1.** Univariate and multivariate regression analyses including the CPET parameters (HR_rec1_, HR_rec3_, HR_max_ and VO_2peak_) as dependent variables and MDS‐UPDRS I.I, step counts, age, sex and beta blocker usage as independent variables. Step counts were divided by 1000 for ease of interpretation. CPET, cardiopulmonary exercise test; HR_rec1_, heart rate 1 min posttest termination; HR_rec3_, heart rate 3 min posttest termination; SCOPA‐AUT, scales for outcomes in Parkinson's disease‐autonomic dysfunction; VO_2peak_, peak oxygen consumption.

**Table S2.** Descriptive characteristics for analysis sample and split for valid and invalid cardiopulmonary exercise test according to the criteria by Thrue et al (11) Mean (standard deviation) or frequency (%) is reported. MD‐UPDRS, movement disorders society‐unified Parkinson's disease rating scale. #Data on symptom onset was missing for one participant that performed a valid test according to Thrue, use of beta blockers was missing for one participant that performed an invalid test according to Thrue. Data on Hoehn and Yahr stage was missing for three participants that performed an invalid test according to Thrue.

**Table S3.** Inclusion criteria Thrue et al (11) Number of tests meeting each criterion described by Thrue et al for the total study sample and split for men and women. APHRM, age‐predicted heart rate maximum; HR_max_, heart rate maximum.

## Data Availability

Data will be shared after publication of the STEPWISE trial.
