# Supplementary material for: Cardiorespiratory Response to Exercise in Parkinson's Disease: Associations with Autonomic Dysfunction and Physical Activity
Source: Mov Disord Clin Pract. 2025 Jun 9;12(11):1882–90. doi: 10.1002/mdc3.70172 (PMC12625118; doi:10.1002/mdc3.70172)
Supplement: Supplementary file 1 — Data S1. Methods and results of MDS‐UPDRS I.I as independent variable. [file MDC3-12-1882-s010.docx]

## Supplementary Material 1

**Methods**

Here, we report an analysis on the self-reported first factor of the MDS-UPDRS I (Movement Disorders Society-Unified Parkinson Disease Rating Scale) on non-motor aspects of experiences in daily living (MDS-UPDRS I.I).^1^ We studied MDS-UPDRS I.I since it predominantly consists of autonomic symptoms and explains 33% of the total variance in the MDS-UPDRS I.

*Statistics*

We explored the relationship between each dependent variable (HR_rec1_, HR_rec3_, HR_max_ and VO2_peak_) and MDS-UPDRS I.I with univariable linear regression. Using multivariable regression, we adjusted for age, sex, beta blocker usage, step count. Step counts were divided by 1000 for interpretation purposes.

**Results**

*Experienced non-motor symptoms in daily life and cardiorespiratory fitness*

None of the multivariable models on cardiorespiratory parameters and the experienced non-motor symptoms in daily life (MDS-UPDRS I.I) showed a statistically significant association, although we observed the same negative trend in HR_max_ as for SCOPA-AUT (MDS-UPDRS I.I; β=-0.80, 95% CI=[-1.81, 0.22]; Supplementary Figure 2 and Supplementary Table 1).

**References**

1. Goetz CG, Tilley BC, Shaftman SR, et al. Movement Disorder Society-Sponsored Revision of the Unified Parkinson’s Disease Rating Scale (MDS-UPDRS): Scale presentation and clinimetric testing results. *Movement Disorders*. 2008;23(15):2129-2170. doi:10.1002/MDS.22340

2. Thrue C, Hvid LG, Gamborg M, Dawes H, Dalgas U, Langeskov-Christensen M. Aerobic capacity in persons with Parkinson’s disease: a systematic review. *Disabil Rehabil*. 2023;45(15):2409-2421. doi:10.1080/09638288.2022.2094480
