# Supplementary figures and images for: Cardiorespiratory Response to Exercise in Parkinson's Disease: Associations with Autonomic Dysfunction and Physical Activity
Source: Mov Disord Clin Pract. 2025 Jun 9;12(11):1882–90. doi: 10.1002/mdc3.70172 (PMC12625118; doi:10.1002/mdc3.70172)

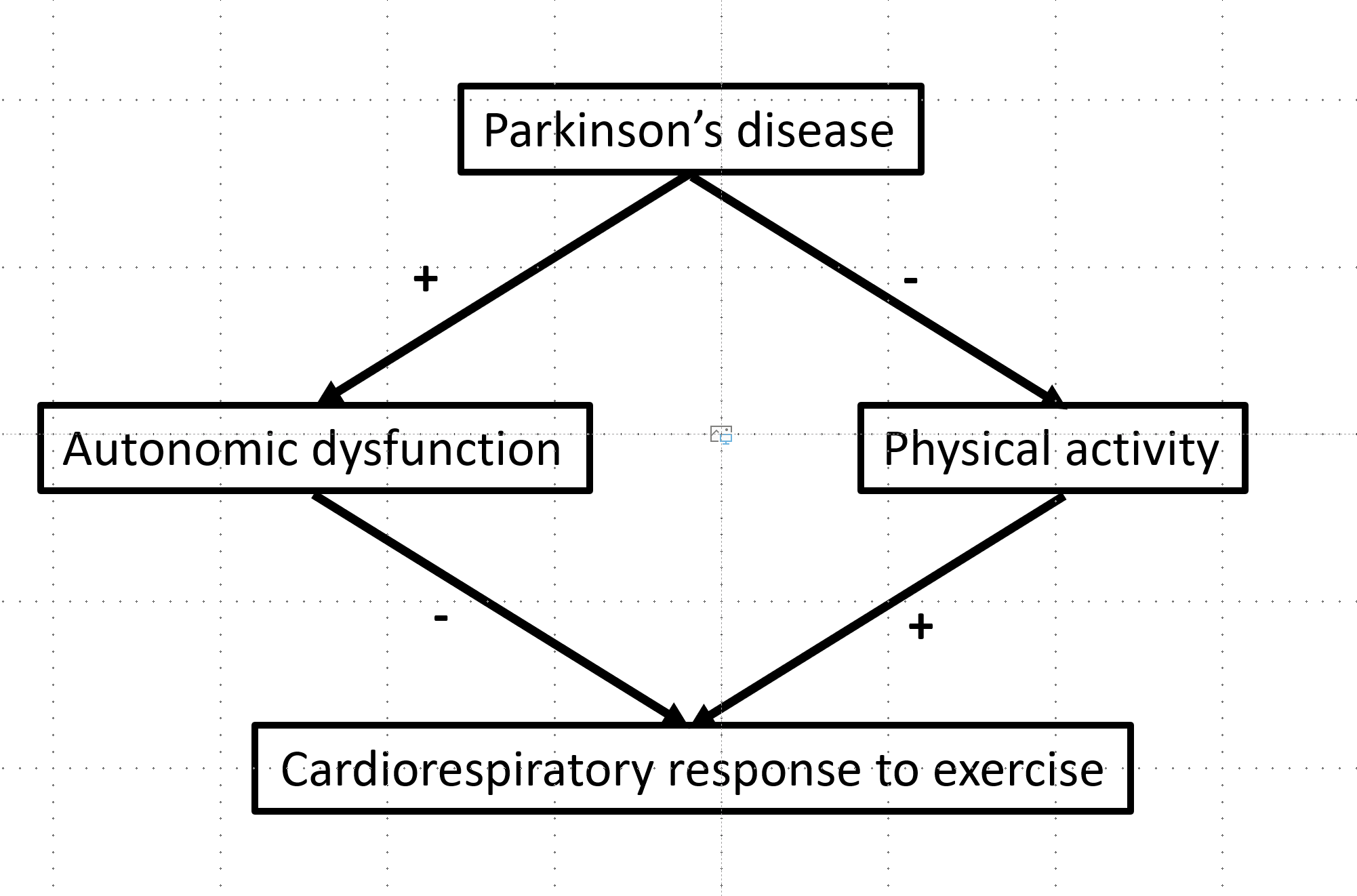

Supplement: Supplementary file 2 — Figure S1. Directed Acyclic Graph of the parameters under study. We hypothesized that Parkinson's disease could lead to autonomic dysfunction, and that autonomic dysfunction reduces the cardiorespiratory response to exercise. We also expect that Parkinson's disease reduces physical activity, and that physical activity results in a reduced cardiorespiratory response to exercise. [file MDC3-12-1882-s003.png]

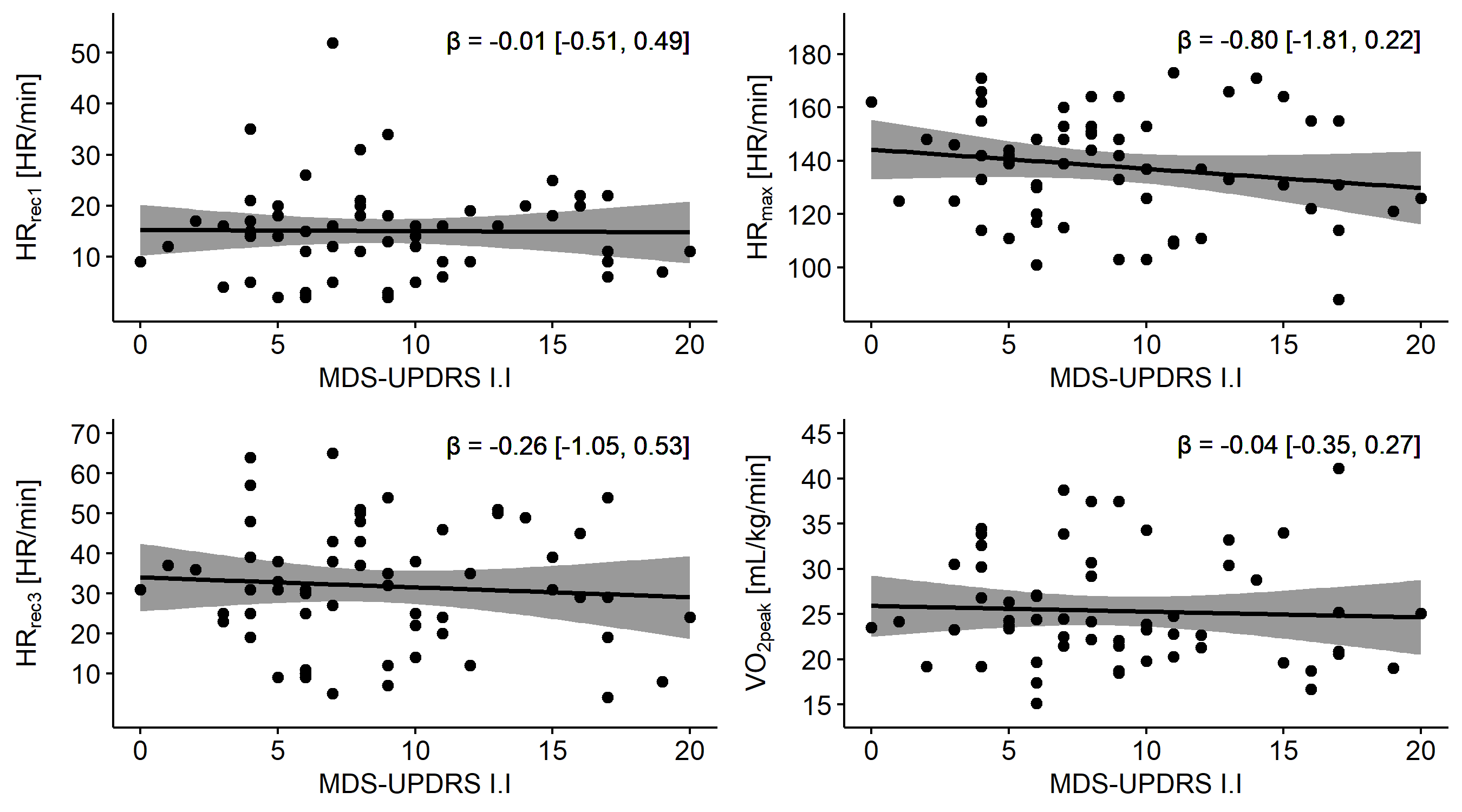

Supplement: Supplementary file 3 — Figure S2. Multivariable regression results of CPET parameters and MDS‐UPRS‐I.I. (A) Heart rate recovery 1 min (HRrec1). (B) Maximum heart rate (HRmax). (C) Heart rate recovery 3 min post exercise (HRrec3). (D) Peak oxygen consumption (VO2peak). We report the beta‐coefficients and 95% confidence intervals. Each data point was corrected for the following covariates: age, sex, use of beta blockers, and step count (divided by 1000). CPET, cardiopulmonary exercise test; MDS‐UPDRS, movement disorders society‐unified Parkinson disease rating scale. [file MDC3-12-1882-s011.png]

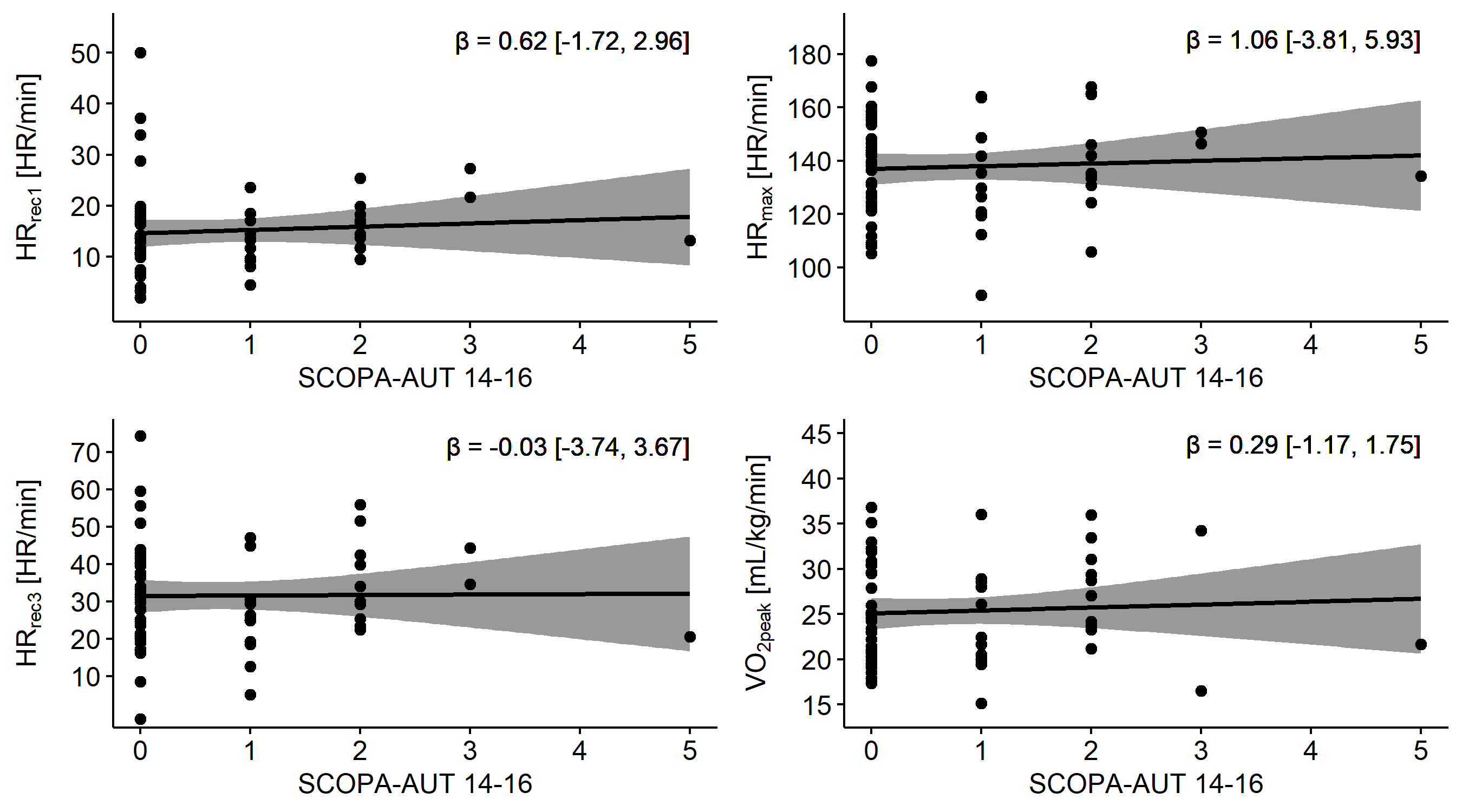

Supplement: Supplementary file 4 — Figure S3. Multivariable regression results of CPET parameters and autonomic dysfunction on items 14–16 of the SCOPA‐AUT. (A) Heart rate recovery 1 min (HRrec1). (B) Maximum heart rate (HRmax). (C) Heart rate recovery 3 min post exercise (HRrec3). (D) Peak oxygen consumption (VO2peak). We report the beta‐coefficients and 95% confidence intervals. Each data point was corrected for the following covariates: age, sex, use of beta blockers, and step count. CPET, cardiopulmonary exercise test; SCOPA‐AUT, scales for outcomes in Parkinson's disease‐autonomic dysfunction. [file MDC3-12-1882-s013.png]

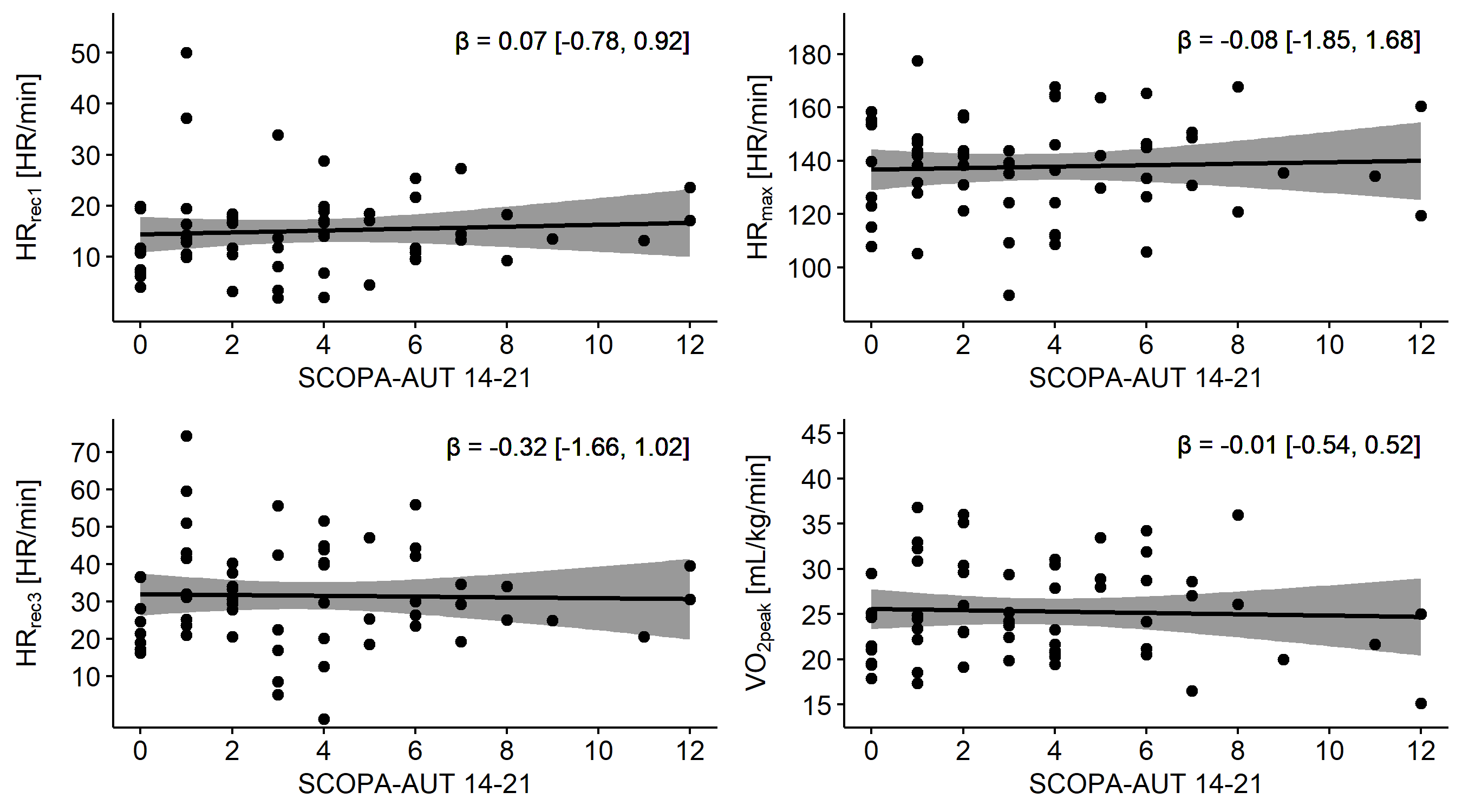

Supplement: Supplementary file 5 — Figure S4. Multivariable regression results of CPET parameters and autonomic dysfunction on items 14–21 of the SCOPA‐AUT. (A) Heart rate recovery 1 min (HRrec1). (B) Maximum heart rate (HRmax). (C) Heart rate recovery 3 min post exercise (HRrec3). (D) Peak oxygen consumption (VO2peak). We report the beta‐coefficients and 95% confidence intervals. Each data point was corrected for the following covariates: age, sex, use of beta blockers, and step count. CPET, cardiopulmonary exercise test; SCOPA‐AUT, scales for outcomes in Parkinson's disease‐autonomic dysfunction. [file MDC3-12-1882-s009.png]

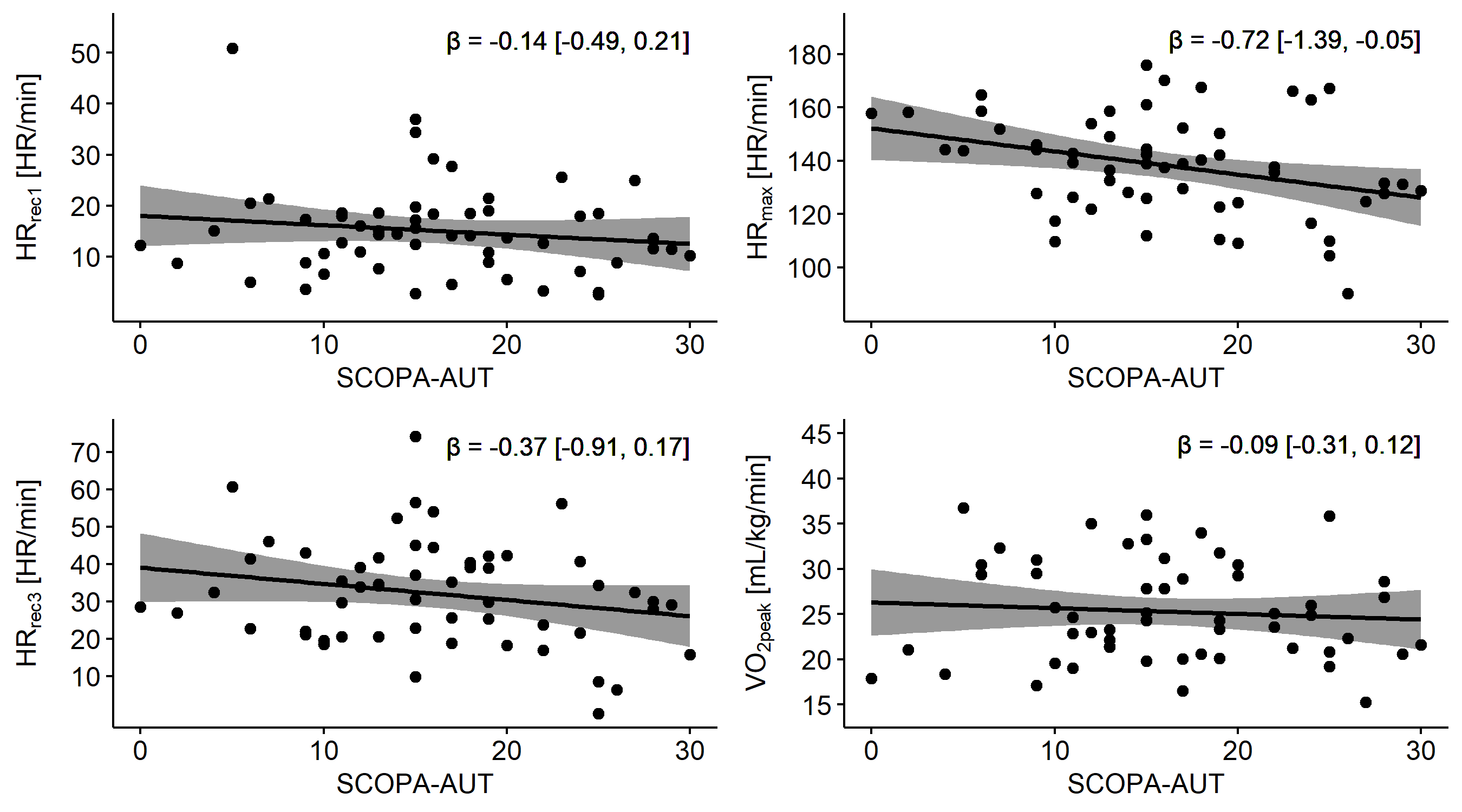

Supplement: Supplementary file 6 — Figure S5. Multivariable regression results of CPET parameters and autonomic dysfunction, excluding three participants on beta blockers. (A) Heart rate recovery 1 min (HRrec1). (B) Maximum heart rate (HRmax). (C) Heart rate recovery 3 min post exercise (HRrec3). (D) Peak oxygen consumption (VO2peak). We report the beta‐coefficients and 95% confidence intervals. Each data point was corrected for the following covariates: age, sex and step count. CPET, cardiopulmonary exercise test; SCOPA‐AUT, scales for outcomes in Parkinson's disease‐autonomic dysfunction. [file MDC3-12-1882-s012.png]

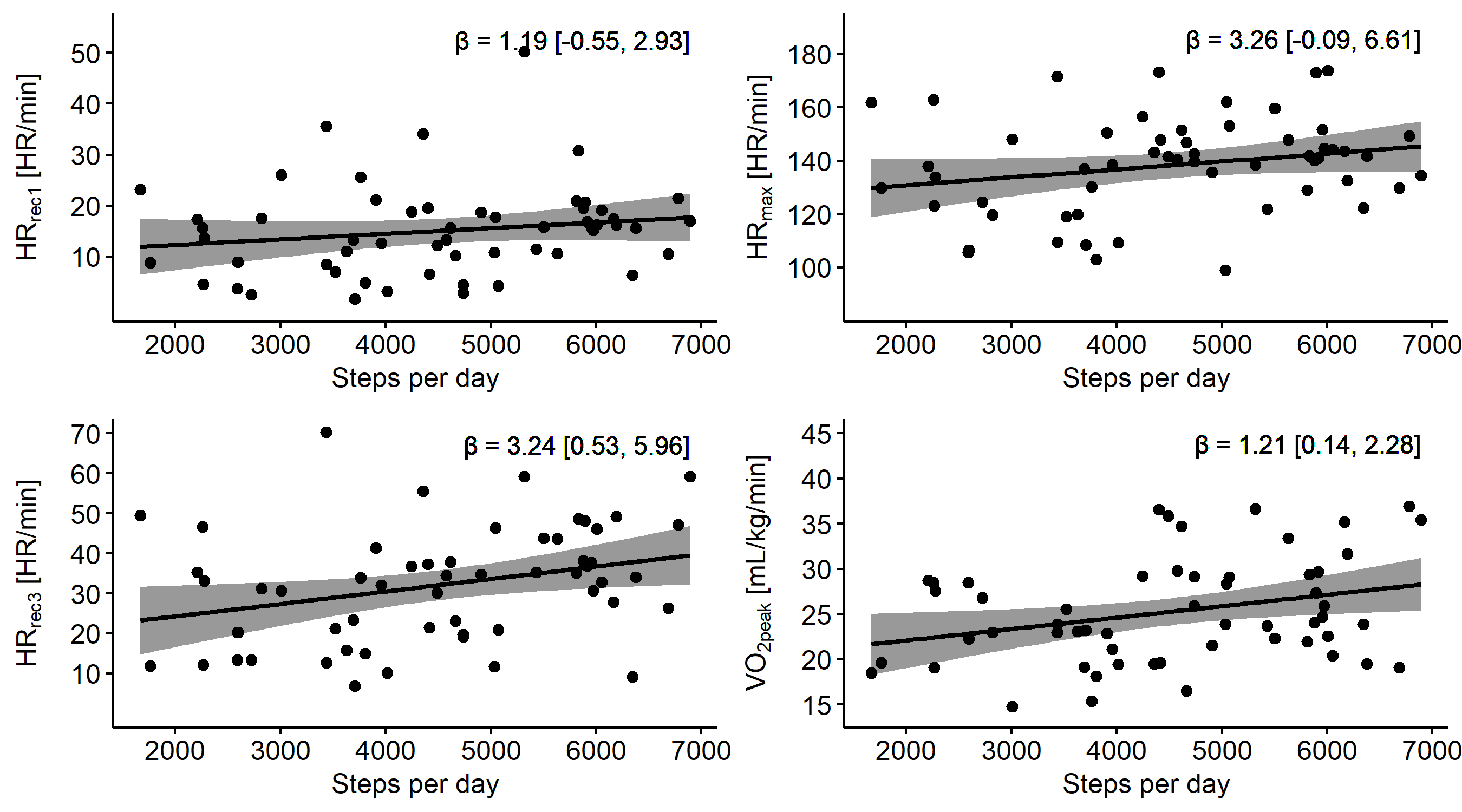

Supplement: Supplementary file 7 — Figure S6. Multivariable regression results of CPET parameters and physical activity, excluding three participants on beta blockers. (A) Heart rate recovery 1 min (HRrec1). (B) Maximum heart rate (HRmax). (C) Heart rate recovery 3 min post exercise (HRrec3). (D) Peak oxygen consumption (VO2peak). We report the beta‐coefficients and 95% confidence intervals. Each data point was corrected for the following covariates: age, sex and scales for outcomes in Parkinson's disease‐autonomic dysfunction (SCOPA‐AUT). CPET, cardiopulmonary exercise test. [file MDC3-12-1882-s002.png]

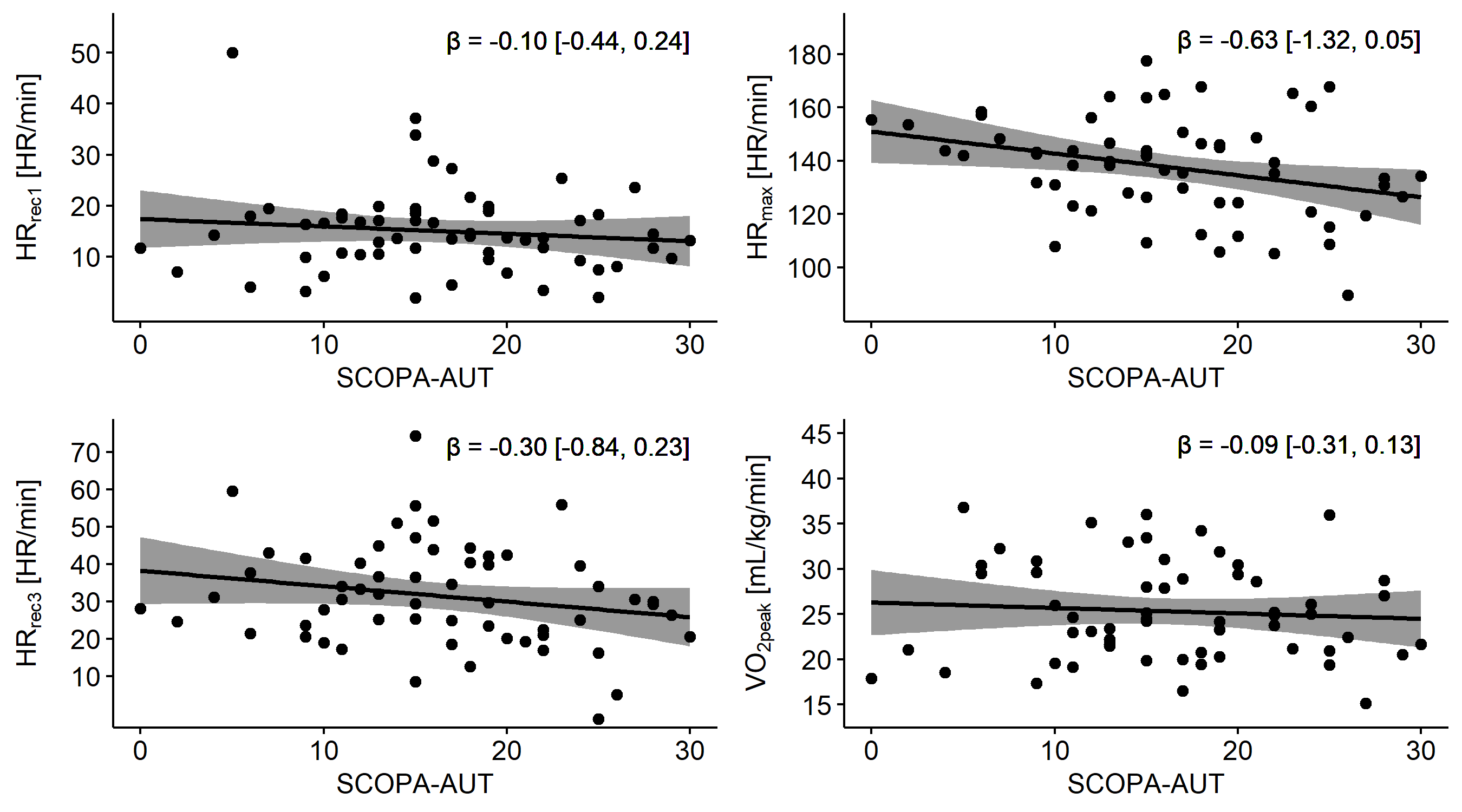

Supplement: Supplementary file 8 — Figure S7. Multivariable regression results of CPET parameters and autonomic dysfunction, correcting for levodopa equivalent daily dose. (A) Heart rate recovery 1 min (HRrec1). (B) Maximum heart rate (HRmax). (C) Heart rate recovery 3 min post exercise (HRrec3). (D) Peak oxygen consumption (VO2peak). We report the beta‐coefficients and 95% confidence intervals. Each data point was corrected for the following covariates: age, sex, step count and levodopa equivalent daily dose. CPET, cardiopulmonary exercise test; SCOPA‐AUT, scales for outcomes in Parkinson's disease‐Autonomic dysfunction. [file MDC3-12-1882-s005.png]

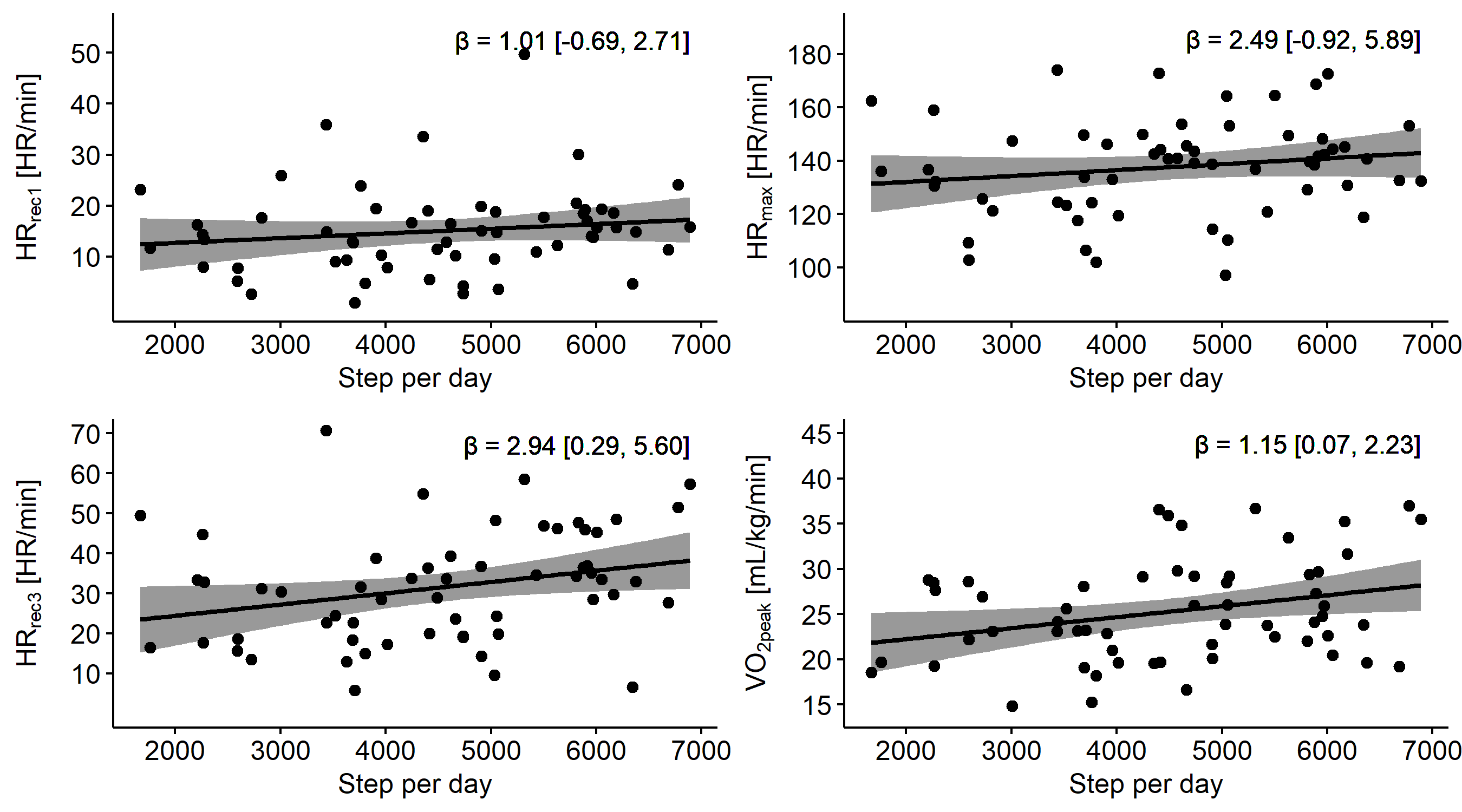

Supplement: Supplementary file 9 — Figure S8. Multivariable regression results of cardiorespiratory parameters and physical activity, correcting for levodopa equivalent daily dose. (A) Heart rate recovery 1 min (HRrec1). (B) Maximum heart rate (HRmax). (C) Heart rate recovery 3 min post exercise (HRrec3). (D) Peak oxygen consumption (VO2peak). We report the beta‐coefficients and 95% confidence intervals. Each data point was corrected for the following covariates: age, sex, SCOPA‐AUT and levodopa equivalent daily dose. CPET, cardiopulmonary exercise test; SCOPA‐AUT, scales for outcomes in Parkinson's disease‐autonomic dysfunction. [file MDC3-12-1882-s007.png]

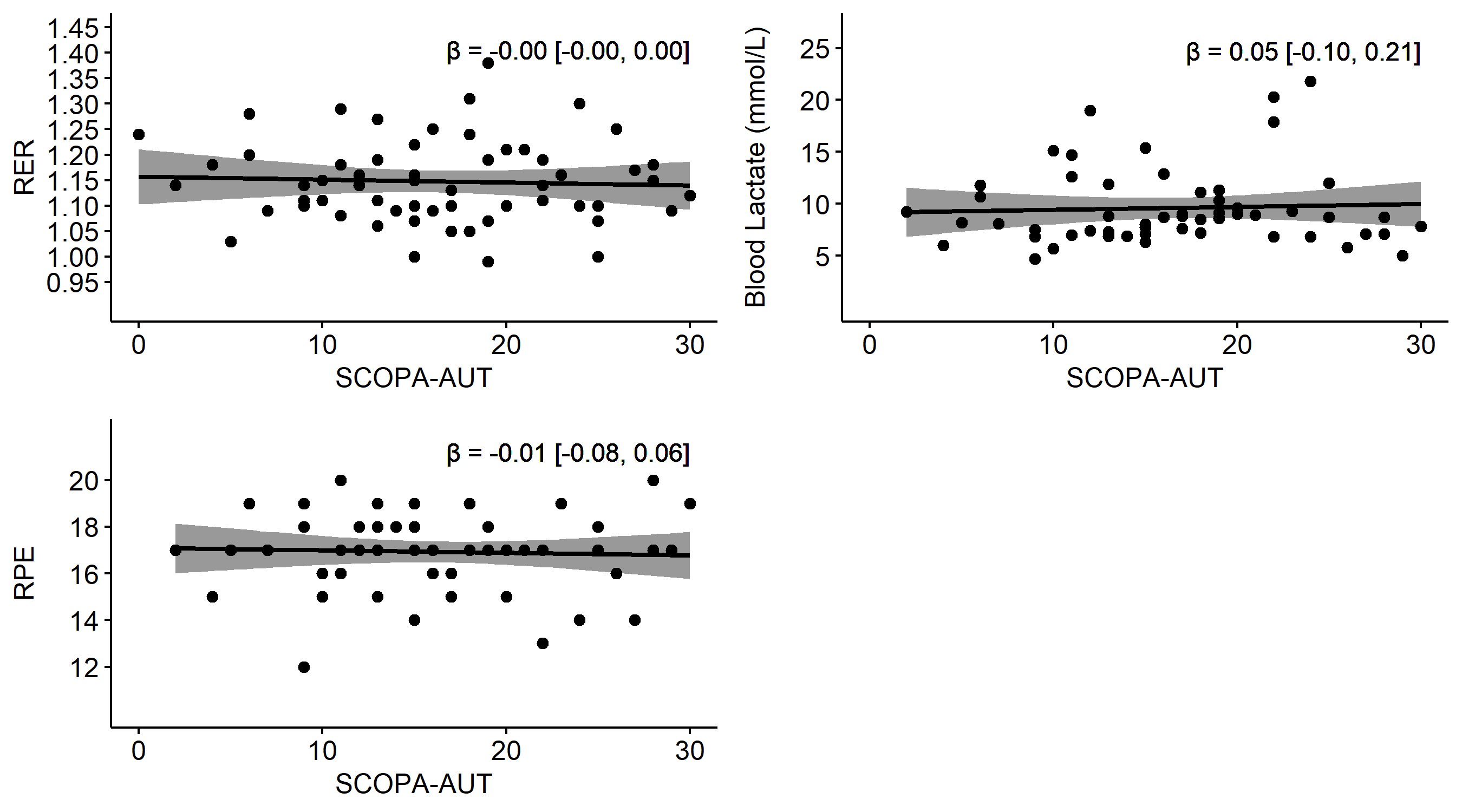

Supplement: Supplementary file 10 — Figure S9. Multivariable regression results including respiratory exchange ratio, blood lactate and rate of perceived exertion at test termination as dependent variables and SCOPA‐AUT as independent variable. We report the beta‐coefficients and 95% confidence intervals. Each data point was corrected for the following covariates: age, sex, use of beta blockers, and step count. RER, respiratory exchange ratio; RPE, rate of perceived exertion; SCOPA‐AUT, scales for outcomes in Parkinson's disease‐autonomic dysfunction. [file MDC3-12-1882-s004.png]
