# Supplementary material for: Cardiorespiratory Response to Exercise in Parkinson's Disease: Associations with Autonomic Dysfunction and Physical Activity
Source: Mov Disord Clin Pract. 2025 Jun 9;12(11):1882–90. doi: 10.1002/mdc3.70172 (PMC12625118; doi:10.1002/mdc3.70172)
Supplement: Supplementary file 11 — Table S1. Univariate and multivariate regression analyses including the CPET parameters (HRrec1, HRrec3, HRmax and VO2peak) as dependent variables and MDS‐UPDRS I.I, step counts, age, sex and beta blocker usage as independent variables. Step counts were divided by 1000 for ease of interpretation. CPET, cardiopulmonary exercise test; HRrec1, heart rate 1 min posttest termination; HRrec3, heart rate 3 min posttest termination; SCOPA‐AUT, scales for outcomes in Parkinson's disease‐autonomic dysfunction; VO2peak, peak oxygen consumption. [file MDC3-12-1882-s008.docx]

| **Dependent**  **variable** | **Independent**  **variable** | **β-coefficient**  **(SE)** | **95%**  **CI** | **p-**  **value** | **Adj r^2^**  **(r^2^)** | **Partial**  **r^2^** |
| --- | --- | --- | --- | --- | --- | --- |
| HR_rec1_ | MDS-UPDRS I.I | -0.02 (0.25) | [-0.52, 0.47] | 0.925 | -0.017 (0.000) | NA |
| HR_rec1_ | Step counts | 1.15 (0.84) | [-0.54, 2.84] | 0.177 | 0.015 (0.032) | NA |
| HR_rec1_ | MDS-UPDRS I.I | -0.01 (0.25) | [-0.51, 0.49] | 0.964 | 0.01 (0.095) | 0.000 |
|  | Step counts | 1.25 (0.85) | [-0.46, 2.95] | 0.149 |  |  |
|  | Age | -0.06 (0.15) | [-0.37, 0.24] | 0.673 |  |  |
|  | Sex | -4.32 (2.51) | [-9.36, 0.72] | 0.091 |  |  |
|  | Beta blocker use | 0.26 (5.51) | [-10.79, 11.32] | 0.962 |  |  |
| HR_rec3_ | MDS-UPDRS I.I | -0.25 (0.42) | [-1.10, 0.60] | 0.563 | -0.012 (0.006) | NA |
| HR_rec3_ | Step counts | 3.26 (1.40) | [0.45, 6.06] | 0.024 | 0.07 (0.087) | NA |
| HR_rec3_ | MDS-UPDRS I.I | -0.26 (0.39) | [-1.05, 0.53] | 0.511 | 0.165 (0.237) | 0.008 |
|  | Step counts | 3.31 (1.34) | [0.62, 5.99] | 0.017 |  |  |
|  | Age | -0.53 (0.24) | [-1.01, -0.05] | 0.032 |  |  |
|  | Sex | -6.31 (3.95) | [-14.23, 1.62] | 0.116 |  |  |
|  | Beta blocker use | -7.08 (8.67) | [-24.48, 10.31] | 0.418 |  |  |
| HR_max_ | MDS-UPDRS I.I | -0.72 (0.56) | [-1.84, 0.40] | 0.205 | 0.011 (0.028) | NA |
| HR_max_ | Step counts | 2.92 (1.92) | [-0.93, 6.77] | 0.134 | 0.022 (0.039) | NA |
| HR_max_ | MDS-UPDRS I.I | -0.80 (0.51) | [-1.81, 0.22] | 0.122 | 0.22 (0.287) | 0.044 |
|  | Step counts | 2.97 (1.73) | [-0.50, 6.44] | 0.092 |  |  |
|  | Age | -0.67 (0.31) | [-1.30, -0.05] | 0.035 |  |  |
|  | Sex | -13.97 (5.11) | [-24.22, -3.73] | 0.008 |  |  |
|  | Beta blocker use | -8.68 (11.21) | [-31.16, 13.8] | 0.442 |  |  |
| VO_2peak_ | MDS-UPDRS I.I | -0.06 (0.17) | [-0.40, 0.28] | 0.710 | -0.015 (0.002) | NA |
| VO_2peak_ | Step counts | 1.27 (0.56) | [0.15, 2.39] | 0.027 | 0.067 (0.083) | NA |
| VO_2peak_ | MDS-UPDRS I.I | -0.04 (0.16) | [-0.35, 0.27] | 0.797 | 0.175 (0.246) | 0.001 |
|  | Step counts | 1.19 (0.53) | [0.13, 2.26] | 0.029 |  |  |
|  | Age | -0.31 (0.10) | [-0.50, -0.12] | 0.002 |  |  |
|  | Sex | 2.51 (1.57) | [-0.64, 5.65] | 0.116 |  |  |
|  | Beta blocker use | -1.28 (3.44) | [-8.17, 5.62] | 0.712 |  |  |

**Supplementary Table 1. Univariate and multivariate regression analyses including the CPET (HR_rec1_, HR_rec3_, HR_max_ and VO_2peak_) as dependent variables and MDS-UPDRS I.I, step counts, age, sex and beta blocker usage as independent variables.**

CPET = cardiopulmonary exercise test; HR_max_ = maximum heart rate; HR_rec1_ = heart rate 1 minute post test termination; HR_rec3_ = heart rate 3 minutes post test termination; SCOPA-AUT = SCales for Outcomes in PArkinson’s disease - Autonomic dysfunction; VO_2peak_ = peak oxygen consumption. Step counts were divided by 1000 for ease of interpretation.
