# Supplementary material for: Cardiorespiratory Response to Exercise in Parkinson's Disease: Associations with Autonomic Dysfunction and Physical Activity
Source: Mov Disord Clin Pract. 2025 Jun 9;12(11):1882–90. doi: 10.1002/mdc3.70172 (PMC12625118; doi:10.1002/mdc3.70172)
Supplement: Supplementary file 12 — Table S2. Descriptive characteristics for analysis sample and split for valid and invalid cardiopulmonary exercise test according to the criteria by Thrue et al (11) Mean (standard deviation) or frequency (%) is reported. MD‐UPDRS, movement disorders society‐unified Parkinson's disease rating scale. #Data on symptom onset was missing for one participant that performed a valid test according to Thrue, use of beta blockers was missing for one participant that performed an invalid test according to Thrue. Data on Hoehn and Yahr stage was missing for three participants that performed an invalid test according to Thrue. [file MDC3-12-1882-s006.docx]

**Supplementary Table 2. Descriptive characteristics for analysis sample and split for valid and invalid cardiorespiratory fitness test according to the criteria by Thrue et al.(11)**

|  | **Total (n=59)** | **Valid Thrue (n=23)** | **Invalid Thrue (n=36)** |
| --- | --- | --- | --- |
| Age [years] | 65.1 (7.9) | 64.5 (8.5) | 65.5 (7.6) |
| Women | 22 (37.3%) | 9 (39.1%) | 13 (36.1%) |
| Body Mass Index [kg/m^2^] | 26.7 (3.9) | 25.6 (3.2) | 27.3 (4.1) |
| Education [years] | 14.0 [12.0-18.0] | 15.0 [13.0-18.0] | 14.0 [12.0-18.0] |
| Disease duration [years] | 4.0 [1.5-6.0] | 3.0 [1.5-5.0] | 4.0 [1.8-7.3] |
| Symptom onset [years]^#^ | 6.5 [4.0-12.0] | 6.0 [3.4-7.8] | 9.5 [4.0-12.1] |
| Hoehn and Yahr stage^#^  1  2  3 | 27 (46.7%)  22 (36.7%)  7 (11.7%) | 12 (52.2%)  7 (30.4%)  4 (17.4%) | 15 (41.7%)  15 (41.7%)  3 (8.3%) |
| LEDD [mg] | 600 [389-818] | 600 [350-840] | 588 [417-814] |
| SCOPA-AUT score | 16.3 (7.0) | 14.6 (5.9) | 17.3 (7.6) |
| VO_2peak_ [ml/kg/min] | 25.3 (6.1) | 28.0 (6.8) | 23.6 (5.0) |
| HR_max_ [bpm] | 137.9 (20.5) | 153.8 (10.5) | 127.7 (18.8) |
| Respiratory Exchange Ratio | 1.15 (0.08) | 1.18 (0.08) | 1.13 (0.07) |
| MDS-UPDRS-III score | 30.7 (11.4) | 27.3 (10.5) | 32.8 (11.6) |
| Six minute walking distance [m] | 494.3 (75.5) | 499.7 (79.4) | 490.9 (73.8) |
| Step counts [steps/day] | 4518.2 (1384.5) | 4736.2 (1239.1) | 4379.0 (1469.7) |
| Current smoker | 1 (1.7%) | 0 (0%) | 1 (2.8%) |
| Use of beta blockers | 3 (5.1%) | 1 (4.3%) | 2 (5.6%) |

Mean (standard deviation), median [interquartile range] or frequency (%) is reported. HR_max_ = maximum heart rate, LEDD = Levodopa Equivalent Daily Dosage, MDS-UPDRS = Movement Disorders Society-Unified Parkinson Disease Rating Scale, SCOPA = SCales for Outcomes in PArkinson’s disease - Autonomic Dysfunction, VO_2peak_ = peak oxygen consumption. #Data on symptom onset was missing for one participant that performed a valid test according to Thrue, use of betablockers was missing for one participant that performed an invalid test according to Thrue. Data on Hoehn and Yahr stage was missing for three participants that performed an invalid test according to Thrue.
