# Supplementary material for: Cardiorespiratory Response to Exercise in Parkinson's Disease: Associations with Autonomic Dysfunction and Physical Activity
Source: Mov Disord Clin Pract. 2025 Jun 9;12(11):1882–90. doi: 10.1002/mdc3.70172 (PMC12625118; doi:10.1002/mdc3.70172)
Supplement: Supplementary file 13 — Table S3. Inclusion criteria Thrue et al (11) Number of tests meeting each criterion described by Thrue et al for the total study sample and split for men and women. APHRM, age‐predicted heart rate maximum; HRmax, heart rate maximum. [file MDC3-12-1882-s001.docx]

**Supplementary Table 3.** **Inclusion criteria Thrue et al.(11)**

|  | **Total (n=59)** | **Female (n=22)** | **Male (n=37)** |
| --- | --- | --- | --- |
| All criteria | 23 | 9 | 14 |
| HR_max_ >90% APHRM | 31 | 14 | 17 |
| Blood lactate (mmol/L) | 58 | 22 | 36 |
| Respiratory Exchange Ratio | 58 | 21 | 37 |
| BORG >=17 | 42 | 13 | 29 |

Number of tests meeting each criterion described by Thrue et al. for the total study sample and split for men and women. HR_max_ = heart rate maximum. APHRM = age-predicted heart rate maximum.
